# Use of Zebrafish Models to Boost Research in Rare Genetic Diseases

**DOI:** 10.3390/ijms222413356

**Published:** 2021-12-12

**Authors:** Lucie Crouzier, Elodie M. Richard, Jo Sourbron, Lieven Lagae, Tangui Maurice, Benjamin Delprat

**Affiliations:** 1 MMDN, University of Montpellier, EPHE, INSERM, 34095 Montpellier, France; lucie.crouzier@umontpellier.fr (L.C.); elodie.richard@umontpellier.fr (E.M.R.); tangui.maurice@umontpellier.fr (T.M.); 2 Department of Development and Regeneration, Section Pediatric Neurology, University Hospital KU Leuven, 3000 Leuven, Belgium; j.sourbron@gmail.com (J.S.); lieven.lagae@uzleuven.be (L.L.)

**Keywords:** animal model, rare diseases, zebrafish, phenotyping, Wolfram syndrome, Dravet syndrome

## Abstract

Rare genetic diseases are a group of pathologies with often unmet clinical needs. Even if rare by a single genetic disease (from 1/2000 to 1/more than 1,000,000), the total number of patients concerned account for approximatively 400 million peoples worldwide. Finding treatments remains challenging due to the complexity of these diseases, the small number of patients and the challenge in conducting clinical trials. Therefore, innovative preclinical research strategies are required. The zebrafish has emerged as a powerful animal model for investigating rare diseases. Zebrafish combines conserved vertebrate characteristics with high rate of breeding, limited housing requirements and low costs. More than 84% of human genes responsible for diseases present an orthologue, suggesting that the majority of genetic diseases could be modelized in zebrafish. In this review, we emphasize the unique advantages of zebrafish models over other in vivo models, particularly underlining the high throughput phenotypic capacity for therapeutic screening. We briefly introduce how the generation of zebrafish transgenic lines by gene-modulating technologies can be used to model rare genetic diseases. Then, we describe how zebrafish could be phenotyped using state-of-the-art technologies. Two prototypic examples of rare diseases illustrate how zebrafish models could play a critical role in deciphering the underlying mechanisms of rare genetic diseases and their use to identify innovative therapeutic solutions.

## 1. Introduction

Rare diseases are commonly defined by their low prevalence in the general population. While there is no worldwide consensus on the definition and, more particularly, a prevalence cut-off number, the European Union stated that any life-threatening or chronically debilitating disease affecting fewer than 1 out of 2000 individuals qualifies as a rare disease. Collectively, these conditions affect around 30 million people in the European Union alone, thus posing a tremendous societal burden. Numerous rare diseases are severe, chronic, mostly genetic in origin and progressive and can be symptomatic as soon as birth or childhood (e.g., infantile spinal muscular atrophy, neurofibromatosis, osteogenesis imperfecta, chondrodysplasias, or Rett syndrome). Others appear only in adulthood (e.g., Huntington’s, Crohn’s, or Charcot–Marie–Tooth’s diseases, amyotrophic lateral sclerosis, Kaposi’s sarcoma and thyroid cancer). There are approximately 8000 rare diseases worldwide and those affected have little hope of treatment due to the lack of knowledge about the pathophysiological mechanism of the disease or the relative lack of private sector investment for therapies that finally concern only few patients and, likely, do not provide a sufficient return on investment. Therefore, it is essential to step up research efforts on rare diseases, at the epidemiological, clinical, genetic and pathophysiological levels, in order to elucidate their biological mechanisms and discover effective therapies. Using powerful technological tools and robust animal models, the pathogenic mechanisms of many rare diseases should be elucidated. It would be important to promote therapeutic research using technological innovations, such as monoclonal antibodies and gene therapy, or even by the discovery of active chemical compounds.

The zebrafish (*Danio Rerio*) is a small tropical freshwater fish species native from Southern Asia and they were first described in 1822 by the Scottish naturalist Francis Buchanan-Hamilton [1]. In recent decades, the zebrafish has become a popular laboratory model, as it bears many significant advantages over other models. This small aquatic vertebrate is easy to maintain in an animal facility and at low costs. Female fish lay a large numbers of eggs per week and the embryos develop quickly and externally through six stages, i.e., embryonic pre-hatching (0–72 h post-fertilization, hpf), post-hatching (72–120 hpf), larval (5–29 days post-fertilization, dpf), juvenile fish (30–89 dpf), adult fish (90 dpf–2 years) and aged fish (from 2 years) [2,3]. In addition, the fully sequenced zebrafish genome [4] reveals that 70% of human protein-coding genes are linked to genes found in zebrafish and 84% of genes known to be associated with human disease have a homologous gene in zebrafish [4]. This renders the model relevant to study genetic disorders linked to human diseases [5,6,7] and particularly neurodegenerative and neurological disorders [8,9,10,11]. To accomplish this, an increasing range of reverse genetic techniques are available to generate new zebrafish models and identify novel genes of interest with potential relevance to human diseases.

Interestingly, several zebrafish organ systems are remarkably similar to those in humans. In addition to neuroanatomical similarities, the zebrafish nervous system also expresses many signaling molecules with a high level of similarity to mammals in terms of signaling pathways, conferring a good descriptive validity in pathophysiological analyses [12,13,14].

Finally, the robustness of their easily quantifiable phenotypes, the ease of treatment with water and a high sensitivity to drugs due to their blood–brain barrier tight junction [15,16], make zebrafish a powerful animal model to identify high-throughput neuroactive compounds. This illustrates the value of this organism as an indispensable model of human neurodegenerative diseases.

In this review, we detail the usefulness of zebrafish lines for the phenotypic analyses of rare genetic diseases and, particularly, exemplify two zebrafish models prototypic for these pathologies, namely, the Wolfram and Dravet syndromes, to help illustrating the relevance for using zebrafish to address pathophysiological mechanisms and drug screening. We also review the tools currently available and their utility in analyzing the zebrafish phenotypes, in particular in the context of defective pathways leading to locomotion, vision, hearing impairments and epileptic phenotypes.

## 2. Zebrafish as a Genetic Disease Model

An animal model gives the prime opportunity to study how genetic and environmental factors can lead to several symptoms related to the disorder.

Zebrafish were initially used in laboratory because of their ability to produce large batches of transparent embryos and to study their embryonic development [17,18,19]. The model allowed researchers to obtain a better understanding of a wide range of cellular mechanisms integrating living organisms such as hepatocarcinogenesis [20], the effects of toxins and alcohol on embryogenesis [21] and tissues and organs regeneration [22,23,24]. Now, zebrafish is commonly used in a variety of biological disciplines ranging from basic developmental biology to applied toxicology. In 2017, more than 1200 laboratories worldwide used this species, in connection with very diverse questions such as the formation and/or regeneration of organs, the intimate functioning of the nervous system, the biological effects of pollutants, or the response to infections, among other examples. Furthermore, the zebrafish is used to model a variety of human diseases, from hereditary muscle diseases [25], neurological disorders [26], cancer [27] and cardiovascular diseases [28] to hematopoietic or infectious diseases [29]. Despite notable differences from human pathophysiology, the zebrafish is a valuable vertebrate model to study vision [30] and hearing disorders [31]. 

### 2.1. Genetic Approaches

In recent years, thanks to innovative technical means [32], it has become possible and relatively easy to mimic human pathologies rapidly and on a large scale in zebrafish by modifying its genome, opening up a very wide field of applications and the search for (novel) therapies. 

#### 2.1.1. Forward Genetics

One strategy, termed forward genetics, is based on the identification of an unknown gene by studying the mutant phenotype of an animal model. The most common technique used to induce mutagenesis consists in exposing germ cells to N-ethyl-N-nitrosourea (ENU), an alkylating agent, that alters, punctually and randomly, the nucleotide sequence of a very large number of genes. Depending on the position within an open reading frame (ORF) or at splicing sites, this can result in a nonsense mutation leading to protein truncation or a missense mutation changing the meaning of the affected codon. ENU treatment can induce loss-of-function and gene inactivation that are transmitted by successive crossings. Then, the thousands of mutants thus generated, each carrying different mutations, are screened to detect potential morphological and functional anomalies [33]. To note, the *didy^s552^* line, a zebrafish model for Dravet syndrome, was discovered through ENU-induced zebrafish mutant bank screening [34].

#### 2.1.2. Reverse Genetics

Powerful reverse genetic methods, such as transient injection of antisense morpholino oligonucleotides (MOs) in embryos, allowed researchers to selectively inhibit gene translation or appropriate splicing and resulted in the transient slaughter of specific genetic products [35,36]. While representing an easy and fast method to generate mutant larvae, the so-called morphants, MOs have disadvantages, including toxicity, incomplete knockdown and occasional off-target nonspecific deleterious effects [37]. Although being relatively stable, MOs become diluted in the animal, implying that their efficacy diminishes at later stages. Therefore, such method is limited to processes occurring during the first 5 days of the fish development, thus excluding certain studies whose phenotypes appear during the juvenile or adult stage. 

RNA interference (RNAi) is a single or double RNA whose interference with a specific mRNA leads to its degradation and to a decrease in the expression of the relevant protein. Insofar as RNA plays a crucial role in gene expression, RNAi blocks it by silencing a particular gene. It is seen as a product of evolution allowing organisms to defend themselves against the introduction of foreign genomes, particularly from viral origin, or even allowing gene expression to be modulated. RNAi by injection has somewhat limited applications, as this approach is restricted to studies of gene function during embryonic development; further, maternally loaded proteins may mask embryonic phenotypes. As of today, this method has not been extensively developed in zebrafish [38].

In order to create gain of function mutant, mRNA injection of synthetic capped mRNA encoding the protein of interest in early embryonic stages (one- or two-cell embryos) is a widely used method. The injected mRNA is distributed more or less evenly with each new cell born from the embryo. The derived mRNA overexpression is a rapid tool for the functional analysis of genes by global expression of gain and loss of function variants of a gene of interest [39,40]. However, this method is limited to the early stages of the embryogenesis process, because, as observed with MOs, the injected mRNA is not long-lastingly stable. 

Other methods of DNA editing, such as the transcription activator-like effector nucleases (TALEN), zinc-finger nucleases (ZFN) or the CRISPR/Cas9 strategy [41,42,43,44], are used to create targeted mutations and to develop stable models of human pathologies [45,46,47,48,49]. The CRISPR/Cas9 technology allows a more precise genome editing, with a site specific insertion of a conditional cassette, to be performed, drastically expending the genetic possibilities [50].

### 2.2. Zebrafish as a Model of Eye Diseases

Vision is among the different functions that can be easily tested using zebrafish models. In fact, the visual system of zebrafish is similar to the human one, with an initial development of the eye structure which, overall, resembles those of other vertebrates [51] but presents some notable structural differences. First, zebrafish eyes are in lateral position with a little overlapped binocular vision, contrary to humans, whose eyes are frontal with highly overlapped binocular vision. In addition, the fovea, the area of the retina where the vision of details is the most precise, is absent in zebrafish. All their optic nerve projections cross at the midline, forming a complete optic chiasm, whereas, in humans, half of the optic fibers project to the ipsilateral side of the tectum [52]. Next, the zebrafish lens is spheroid, extending through the iris and conferring a wide field of vision, compared to humans’ lens, which is ellipsoid. Finally, zebrafish have a tetrachromatic vision, while humans have a trichromatic vision, lacking sensitivity to ultraviolet light [53].

As zebrafish use vision to protect themselves from predators and seek food, their visual system develops rapidly. Similar to the human eye, the retina is composed of different cell types and organized in nuclear layers separated by plexiform layers. Three nuclear layers contain the cell somas, i.e., the outer nuclear layer (ONL), the inner nuclear layer (INL), the ganglion cell layer (GCL) and two plexiform layers, forming an area where axons and dendrites make synapses—the inner plexiform layer and the outer plexiform layer. Photoreceptor (rods and cones) and horizontal cell bodies are located in the ONL, while bipolar, amacrine and Müller glial cells reside in the INL. The GCL is composed of the cell bodies of ganglion cells and their axons constitute the retinal nerve layer to carry the visual information from the eye to the brain [54].

Photoreceptor cells are grouped into rods and cones, arranged in a mosaic pattern in the ONL and specialized for phototransduction. Zebrafish photoreceptor cells have two major subtypes, namely, rods, which are sensitive to light and permit for dim light vision, and cones, subdivided into four classes that include blue-sensitive cones (407–417 nm), green-sensitive cones (473–480 nm), red-sensitive cones (556–564 nm) and UV-sensitive cones (360–361 nm) [55,56]. The latter are involved in vision during bright light as well as color vision. Interestingly, the UV-sensitive cones develop first and are visible in the retina at 4 days post fertilization (dpf). They are followed by short, middle and long sensitive cones. Rod photoreceptors are present at 5 dpf but their contribution is only measured at stages older than 15–21 dpf. The visual system of the zebrafish becomes functional about 3.5 dpf. However, recent studies suggested that rod photoreceptors may be functional at 5 dpf by using a modified visual motor response protocol [57,58]. At this stage, all types of retinal cells are differentiated and the retinal circuits and their projection towards the brain have matured enough to support the first visually triggered motor behaviors, such as visual startle and optokinetic responses [54,59,60]. 

Despite the differences described above, the zebrafish visual system better reflects the human system than any other animal model. Indeed, mice have a vision dominated by rods, whereas, as mentioned previously, zebrafish have a predominantly conical human-like vision. Therefore, the study of human disorders related to cone degeneration are more relevant in zebrafish models. In addition, the visual system develops faster in zebrafish than in mice, this being mature at 5 dpf in zebrafish vs. 15 days of life in mice. Taken together, these facts support the use of zebrafish as an excellent model for understanding human ocular diseases [45,61,62]. 

### 2.3. Measurement of Visual Behavior in Zebrafish

To assess visual functions and their potential alteration in Zebrafish, various tests have been developed. In this review, we focus on the three most popular ones in laboratory practice.

The optomotor response (OMR) is an innate orienting behavior evoked by whole-field visual motion and is common to fish during locomotion, such as when swimming. Thus, the OMR test has been developed as a tool measuring the vision of larvae or adult zebrafish and mediated by the red/green cones pathway [63,64]. 

Movable black-and-white bands revolve around or below the fish that tends to swim in the same direction as the bands. The OMR behavior is a result of the larvae attempting to counter water currents and to remain in place. A control fish with no visual problem swims in the direction of the bands, while a fish with visual impairment swims randomly. The striped pattern can be changed by modulating the contrast of the bands or the wavelength of the OMR. A large number of fish can be tested at the same time, making the OMR test useful for screening at high speed. The assay can be performed as early as 6 dpf, the time of onset of this adaptive behavior [53].

This visual test has been used in genetic screens to identify mutations disrupting the development and function of the visual system [63]. It has been efficiently used to screen molecules as different compounds can be tested with various fish concomitantly or with isolated fish [65].

The *optokinetic response (OKR)* is the eye movement reflex in response to a moving stimulus to help stabilize the image on the retina maintaining visual acuity [66]. This ability develops at 3 dpf [60] and matures significantly by the 5th dpf. This natural response is important for spatial orientation, hunting their prey and escaping from predators.

During the OKR assay, zebrafish larvae are usually immobilized in a methylcellulose solution with eyes keeping the ability to move. A black-and-white striped pattern moves around the fish and the speed of rotation of the bands, their frequency and the contrast can be modified [53]. The eyes of the larvae are pigmented; therefore, their movements can be easily tracked under a binocular magnifier or a camera connected to a computer equipped with tracking software [67]. Eye movements, called saccades, consist of a smooth pursuit (slow phase) in the direction of the rotation of the stimulus and a fast resetting (fast phase) in the opposite direction after the image left the visual field. The number of saccades reflects the quality of visual acuity, with a small number of saccades indicating a visual impairment. The speed, amplitude and duration of the saccades can also be quantified and used as indicators of the visual deficit. Therefore, the optokinetic response can be successfully used to screen visual performance following genetic manipulations and/or drug treatments [65,68].

The *visual motor response (VMR)* is a sensorimotor behavior resulting in a rapid and protective response to sudden stimuli, observed within a couple of seconds following a visual stimulus. This natural response, based on the natural tendency to flee from predators, develops at 72 hpf and is based, more specifically, on the larvae’s ability to detect sudden changes in light [69]. This test is complementary to the OKR and allows researchers to discriminate the ability of the fish to detect movement (=OKR) from changes in light intensity (=VMR).

The locomotor response of each larva, following a controlled change in white light, is quantified by an automated tracking system. The movement is quantified as a number of video pixels changing beyond a predefined threshold in successive images. This frame-by-frame movement can be averaged over a specific period of time. In control fish, the locomotor activity increases drastically following a light–dark transition and returns to the baseline when the dark–light transition takes place. Thus, the difference in reaction caused by the change in brightness can be measured and compared as a function of the different genotypes tested [69].

The VMR test can be used as a powerful tool for a high-throughput in vivo screening platform for pharmacologically active molecules [68,70]. Indeed, a large number of fish can be tested simultaneously. Automated quantification and execution ensure reproducible results, which are key in drug screening assays. 

### 2.4. Zebrafish as a Model of Deafness

Numerous studies have highlighted that the zebrafish is also an excellent model for hearing and balance disorders [71,72,73,74], hair cell death and regeneration, ototoxicity and drug screening [75,76,77,78,79].

One specific advantage of using zebrafish is the conservation, in vertebrates, of the anatomy and physiology of the inner ear structures [80,81]. Most particularly, the anatomical characteristics of the vestibular labyrinth are highly conserved in terms of structure and function. The vestibular system, composed of two otolithic organs (saccule and utricle) and three semicircular canals (horizontal, vertical anterior and posterior canal), maintaining the body balance. The detection of linear horizontal acceleration occurs in the utricle and the semicircular canals detect angular acceleration in different planes. The saccule is used, in humans, for the detection of vertical acceleration. However, in zebrafish, the saccule is thought to be primarily responsible for sound detection, with frequencies between 200 Hz and 4000 Hz. 

The inner ear of mammals comprises also the cochlea, a bony labyrinth filled with fluid, which transduces sound stimuli. It contains the organ of Corti, consisting of about 20,000 sensory receptors, called hair cells because of their protruding stereocilia bundles. The cochlea and the vestibular system are connected to the brain by the eighth cranial nerve (vestibulocochlear). One branch of this nerve, the auditory nerve, transmits sound signals to the brain and another transmits signals related to balance. 

Fish do not have a dedicated auditory organ such as the mammalian cochlea, but their inner ear is made of three chambers, i.e., the saccule and the lagena, which are necessary for auditory perception, and the utricle, which is essential for postural equilibrium [82,83,84]. These compartments, filled with liquid, are attached, at their posterior end, to four small bones, the Weberian ossicles, which are interconnected by ligaments and form a connection to the swim bladder. The Weberian ossicles function as an accessory hearing structure transmitting sound vibrations from the swim bladder to the sensory organs of the inner ear [85].

The semicircular canals are also present with the same functions as in humans. They are lined with connected hair cells that bathe in a fluid with high potassium concentration called endolymph. In the presence of vibrations due to sound, the cells vibrate and send a nervous message to the brain [86]. The morphological, electrophysiological, biochemical and molecular characteristics of hair cells are largely preserved from fish to human. A number of genes necessary for hair cell development and function in zebrafish have been shown to be associated with hearing loss in mice and humans, revealing their conserved function [83].

In some species, the swim bladder is connected to the inner ear either directly through small canals or via a chain of bones and transmits high-frequency sounds. Each of these compartments contains an otolith, a crystalline structure of calcium carbonate (CaCO_3_) involved in the body balance. Each fish has three otoliths, namely, the lapillus, sagitta and asteriscus. During movement or vibration, otoliths exert pressure on the ciliary bundles of macular hair cells, which become deflected and send signals to the brain. These signals are used for hearing or balancing, in fish.

In addition to the ear, zebrafish possess another mechanosensory system that employs sensory hair cells, the lateral line organ. It encodes hydrodynamic information required for fundamental behaviors, including rheotaxis [87,88,89], schooling behavior [90], prey detection [91,92,93] and predator avoidance [94].

The lateral line contains hair cells and supporting cells in sensory patches, on the surface of the epidermis or inside epidermal canals, called neuromasts. Arranged in precise lines over the body surface, each neuromast is innervated by two bipolar neurons located in a cranial ganglion and establishes its central projection in the rhombencephalon, whence sensory information is transmitted to the brain [95]. The stereocilia bundles of the sensory cells are embedded in a gelatinous cupula which projects into the water and is deflected by the stream of water [96]. Due to the similarities between human hair cells and zebrafish neuromasts, great attention was given to their development [97,98], as well as their regenerative capacity, which distinguishes them from the hair cells of the human ear [99].

### 2.5. Measurement of Hearing Behavior in Zebrafish

Similar to the hair cells in the inner ear of mammals, hair cells in the zebrafish lateral line are killed by exposure to chemicals, including aminoglycosides and cisplatin [99,100,101]. The ease of visualization—thus of quantification as well as the cellular and molecular properties shared with the hair cells of the inner ear—renders this system a good model to study the genetic and pharmacological modulation of the sensitivity of hair cells to potentially ototoxic agents [102]. In recent years, induced ototoxicity of the lateral line of zebrafish testing has become a powerful biological model system to develop new drugs to halt or prevent hearing loss. Among other studies, Domarecka et al. [103] and Vlasits et al. [104] used the zebrafish lateral line to screen a library of repurposing Food and Drug Administration (FDA)-approved drugs (Enzo 640) and identified two therapeutic drugs, paroxetine and benzamil, which protected against cisplatin-induced hair cell death. 

At 5 dpf, zebrafish have a functional hearing system comparable to that of mammals [105] and can initiate an escape response to a sudden acoustic stimuli [106,107]. The characteristics of the zebrafish auditory system facilitates the study of the effects of noise on the inner ear and behavioral response pathways. The *acoustic startle response (ASR)* is a muscular activity, produced by reflex in response to a more or less loud and sudden sound, that is easy to measure and quantify [108]. As for the VMR test, following a sound stimulus, the ASR measures the locomotor response of each larva in a 96-well plate using an automated tracking system. In a physiological condition, the locomotor activity of control larvae increases strongly following the sound stimulus and returns to a baseline in the absence of sound. In a pathological condition, the reaction of mutant larvae or larvae previously treated with ototoxic agents to the sound stimulus is altered. The difference in reaction can be objectively measured and compared as a function of different conditions tested [109].

### 2.6. Zebrafish as a Model of Epilepsy

Epilepsy is a relatively prevalent neurological disease affecting nearly 70 million people worldwide and is characterized by recurrent seizures [110]. Even though patients with epilepsy (PWEs) are a heterogeneous group, they share the excessive neuronal excitation during a seizure; this can be caused by alterations in (i) inhibitory and/or excitatory neurotransmission or (ii) gene expression encoding proteins modulating neuronal activity, e.g., ion channels such as the sodium voltage-gated channel alpha subunit 1 (SCN1A). The International League Against Epilepsy (ILAE) has made the following etiological classification into six subgroups: (1) structural cause, (2) genetic mutation, (3) metabolic defect, (4) abnormal immune reaction, (5) infection and (6) unknown [111]. In nearly 30% of the PWEs, the cause is unknown. Structural anomalies (e.g., traumatic brain injury, tumor, or stroke) and infection (e.g., encephalitis) are among the most common causes [112].

Even though zebrafish models offer prodigious possibilities to mimic these causes, there is still an unmet need to investigate epileptogenesis, mechanistic pathways and novel anti-seizure medication (ASM) in these zebrafish models [112]. 

In contrast, genetic causes of epilepsy have been extensively studied in zebrafish, partially due to the fact that 80% of the known epilepsy-related genes are found in the zebrafish genome [113,114,115,116,117]. Interestingly, developmental and epileptic encephalopathies (DEE), due to genetic defects, are more common in younger PWEs [118] and, in some PWEs, one single gene can be causative for the epileptic phenotype [119]. Thanks to the powerful, ever-expanding and effective techniques for zebrafish genome manipulation, we are able to generate zebrafish epilepsy models, recapitulating the main characteristics of the human disease [112,120,121]. Albeit current epilepsy research has been focusing on these genetically engineered zebrafish, the first zebrafish epilepsy model was induced by chemicals [122] and chemically induced zebrafish seizure models are still used nowadays for studying epileptogenesis and discovering novel ASM, e.g., by using pentylenetetraz-ole (PTZ) and ethyl ketopentenoate (EKP) [123,124,125,126,127].

### 2.7. Measurement of Seizures in Zebrafish

Zebrafish larvae are mostly used as a model for epilepsy and seizures due to several advantages, such as high fecundity, leading to numerous fast-developing embryos ex utero allowing researchers to perform easy follow-ups of development and genetic manipulations; possibility for high-throughput analyses using larvae, where each can fit in one well of a 96-well plate; minimal ethical regulations; and relatively low costs for maintenance and housekeeping [121]. However, adult zebrafish are sometimes preferred due to a higher developed neural system, easier handling and certain parameters that sometimes cannot be observed in larvae, e.g., distinct seizure stages [128,129].

#### 2.7.1. Two-Stage Locomotor and Electrophysiological Setup

The most accurate and preferred seizure characterization and ASM screening strategy encompasses a two-stage locomotor and electrophysiological setup in zebrafish larvae, rather than adult zebrafish, due to the aforementioned advantages of these larvae [120,121,123].

The locomotor activity is a behavioral assay usually assessed using an automated tracking system in which a 96-well plate can be fitted. Different locomotor read-outs can be obtained, such as total movement [130], total distance in large movements [131] and distance travelled and mean velocity of swim movement [132]. As an illustration, the significantly higher locomotor activity in genetically manipulated mutants than wildtype controls can indicate seizure-like activity. The 96-well plate allows an easy repetition of experiments to be performed; further, diverse compounds can be tested in one single experiment, always compared to a proper control. A decrease in locomotor activity elicited by a certain compound, compared to the vehicle-treated controls, suggests an antiseizure effect. Similar to experiments in rodent epilepsy models, it is of utmost importance to use a sufficiently large group of controls and treated larvae. In addition, one should repeat the locomotor experiment at least once to avoid biological variation among other sources of variation [126,131,133,134,135,136]. Unfortunately, this statically appropriate strategy [137] has not been established in each zebrafish laboratory (e.g., [138]). Moreover, some researchers that focus on ASM discovery only perform locomotor experiments without any electrophysiological confirmation (e.g., [139]), which can lead to false positives, for example, in the case of sedatives or muscle-relaxants, and underlines the need to confirm an antiseizure effect by electrophysiological experiments. These experiments usually enclose local field potential (LFP) recordings by which brain activities are recorded from a single electrode in a small part of the brain [126,140]. However, LFP recordings have some limitations, since there is no spatiotemporal resolution and a relatively long duration (10 min) is needed for each recording per zebrafish larva, which contrasts with the high-throughput locomotor experiments. Therefore, several research groups [112,126,127,141,142,143] have explored other strategies to record brain activity in a relatively easier, more straight-forward and faster manner, as depicted in Table 1.

Even though we underline that the above-mentioned paired selection criterion (locomotor and brain activity assays) is validated in zebrafish epilepsy research, it is different from the clinical setting where ASM can suppress seizures without reducing epileptic events on the electroencephalogram [144,145].

#### 2.7.2. iZAP and Multi-Electrode Array

The *integrated Zebrafish Analysis Platform* (iZAP), constructed by Hong et al. [142] is a novel multichannel electrophysiology unit that can measure brain activities of multiple zebrafish larvae at once. Additionally, there are several loading chambers that allow treatment to be administered over several days and replenishment of the medium to be performed. Furthermore, four extra electrodes per zebrafish larva can record other electrical events, i.e., electro-encephalography, electrooculography, electromyography and audiology. With this in mind, Meyer et al. [143] developed a new microarray recording method, which makes it possible to record up to 61 locations of the zebrafish larval head. This method allows multiregional, synchronous (seizure) brain activity to be detected and can be used for long-term recordings up to ten days.

#### 2.7.3. Bioluminescence and Fluorescence Calcium Imaging

Neuronal activity can be assessed in freely swimming zebrafish larvae using bioluminescence [146]. Transgenic zebrafish expressing green fluorescent protein (GFP)-apoAequorin (Ca^2+^-sensitive bioluminescent photoprotein) under the control of the elavl3 promoter exhibit neuronal expression of apoAequorin (transgenic *Tg(elavl3:eGFP-apoAequorin)* zebrafish). After 24 h coelenterazine treatment, the luminescence intensity corresponds to the brain activity. This approach has been validated by the proconvulsant, EKP, that resulted in a statistically significant increase in the average light signal. EKP acts as an inhibitor of glutamic acid decarboxylase, thereby hampering the conversion from glutamine into γ-aminobutyric acid (GABA), which results in relatively more excitation [127]. Other researchers, using a different transgenic zebrafish model (*Tg(elavl3:GCaMP6s)*), demonstrated that genetically encoded calcium indicators combined with two-photon imaging could accurately report epileptic brain activities [112,141]. Accordingly, it is feasible to outcross the aforementioned transgenic zebrafish with any genetic zebrafish model of epilepsy to examine epileptiform brain activities. 

## 3. The Wolfram Syndrome (WS)

### 3.1. Physiopathology of the WS

**Figure 1:**
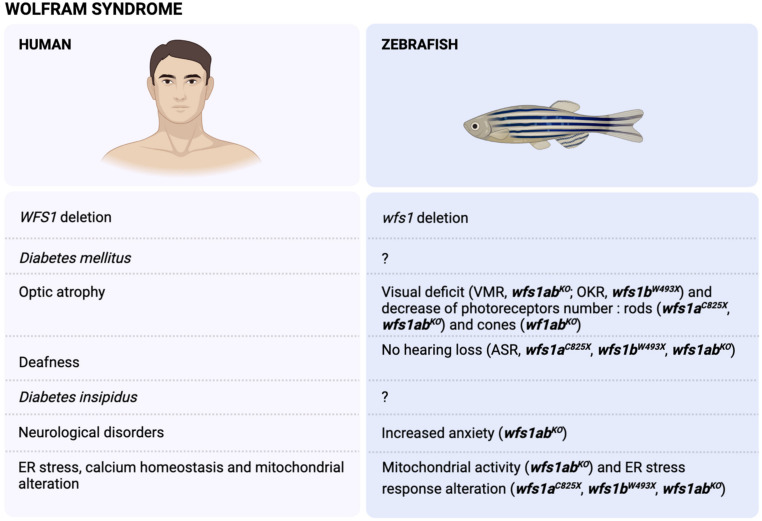
*WFS1* mutant zebrafish as useful tool to decipher physiopatological deficits induced in Wolfram syndrome patients. The *WFS1* gene mutation causes Wolfram syndrome, which is correlated with four main symptoms, i.e., diabetes mellitus, optic atrophy, deafness, diabetes insipidus and neurological disorders. At different cellular levels, these deficits are mainly induced by ER stress response, calcium homeostasis and mitochondrial activity alterations. In zebrafish larvae (5 dpf), these deficits are mostly reproduced; therefore, they mimic human pathology, thus making them a good study model for Wolfram syndrome. Adapted from references [147,150,151,185,186].

The Wolfram syndrome (OMIM #222300) was first described by Wolfram and Wagener [147], who reported four juvenile-onset diabetes with optic nerve atrophy. The acronym DIDMOAD [148] summarizes the most frequent symptoms, i.e., *Diabetes Insipidus*, *Diabetes Mellitus*, optic atrophy and deafness. Additional symptoms include renal and vesical dysfunctions [149], peripheral neuropathy [150], mental retardation and psychiatric illness [151] (Figure 1). This pathology is fatal and death occurs at the median age of 35 years, with severe neurological disabilities, including apneic spells, organic brain syndrome or dementia, or bulbar dysfunction. Death is most often due to central respiratory failure [152]. 

WS is a very rare autosomal-recessive disease. Its prevalence is 1/770,000 [148], with an extremely high heterogeneous prevalence among populations—1/500,000 in the pediatric population of the United Kingdom [153], 1/710,000 in Japan [154], 1/100,000 in North America [155] and 1/68,000 in Lebanon [156]. Two types of this genetic disorder have been identified, Wolfram syndrome 1 (*WFS1*) and Wolfram syndrome 2 (WFS2) [157,158]. The classical form of WS is caused by mutations of both alleles of the nuclear *WFS1* gene, located on chromosome 4p16.1. The gene of 8 exons (33.4 Kb of genomic DNA) encodes a transmembrane protein of 890 amino acids called Wolframin, localized in the endoplasmic reticulum (ER) [158,159,160,161]. Wolframin is a hydrophobic and tetrameric protein with nine transmembrane segments and large hydrophilic regions at both termini [162]. Similar to the many membranous ER proteins [163,164], the localization of *WFS1* facilitates its function as a component of the unfolded protein response (UPR). It also maintains ER homeostasis, notably in pancreatic β-cells [165], by inducing cation channel activity on ER membranes [166] and regulating calcium levels in ER [167,168]. 

*WFS1* is rather ubiquitously expressed in human adults, in a variety of tissues such as heart, brain, placenta, lung, liver, skeletal muscle, kidney and pancreas [158,159]. In mice brain, *WFS1* gene expression levels are higher in brain structures related to emotions or learning and memory, as shown by a very strong expression of *WFS1* in central amygdala and ventral striatum. A strong *WFS1* expression was also detected in the hippocampal CA1 region, parasubiculum, the superficial part of the second and third layers of the prefrontal cortex and proisocortical areas, hypothalamic magnocellular neurosecretory system and central auditory pathway. *WFS1* expression has been detected in numerous brainstem nuclei and in laminae VIII and IX of the spinal cord. *WFS1*-positive nerve fibers were found in the medial forebrain bundle, reticular part of the substantia nigra, globus pallidus, posterior caudate putamen, lateral lemniscus, alveus, fimbria, dorsal hippocampal commissure, subiculum and in the central sublenticular extended amygdala, compact part of substantia nigra and ventral tegmental area [169].

Alterations in its quality or quantity are at the origin of many human pathologies, such as certain types of diabetes or neurodegenerative diseases. Indeed, *WFS1*-deficiency increases endoplasmic reticulum stress, impairs cell cycle progression and triggers the apoptotic pathway specifically in pancreatic β-cells, leading to diabetes mellitus [165,170,171,172,173]. *WFS1*-deficient mice recapitulate several aspects of the neurological manifestations of WS, such as impaired behavioral adaptation to stress including elevated levels of serum corticosterone upon exposure to stress [174,175,176], stress-induced depressive behavior [177] and alterations in visual function, especially the retina [178].

El-Shanti et al. [179] identified a potential second locus, designed WS2 (OMIM #604928), which maps to chromosome 4q22–24. This disorder is due to mutations of the *CISD2* gene [157]. The CISD2-encoded protein, an ER intermembrane small protein (ERIS), is a zinc finger that localizes to the ER and regulates the UPR and Ca^2+^ homeostasis, as well as autophagy [157,180]. Patients with WS2 gene mutation develop the same symptoms except diabetes insipidus [181]. However, they present other symptoms, such as profound upper gastrointestinal ulceration, bleeding and defective platelet aggregation [182,183,184].

### 3.2. Modelling of WS in Zebrafish

As the zebrafish genome is duplicated, the genome contains two different genes, *WFS1a* and *WFS1b*. Therefore, two mutants have been generated by ENU mutagenesis. The first line, *WFS1a^C825X^*, has the *WFS1a* gene invalidated by the replacement of a cysteine by a stop codon at position 825. The second line, *WFS1b^W493X^*, has the *WFS1b* gene invalidated by the replacement of a tryptophan by a stop codon at position 493. A third mutant line was generated from crossing the two lines to generate the double mutant *WFS1a^C829X^* × *WFS1b^W493X^*, called thereafter *WFS1ab^KO^* line, for which both *WFS1* genes are invalidated. A first behavioral analysis showed that *WFS1b^W493X^* zebrafish exhibited a decrease of their visual motor response and optokinetic response [185]. The *WFS1ab^KO^* line showed an increased locomotion in visual motor response and in touch escape response, showing visual deficit and/or exacerbated anxiety. Acoustic startle response was unchanged, thus suggesting an absence of hearing loss [186] (Figure 1).

### 3.3. WFS1 and Stress Response

The ER is the compartment in which the proteins are folded with or without the help of chaperone proteins, then matured by post-translational modifications. Signaling pathways are activated when protein folding is inhibited or disturbed and their primary purpose is to decrease protein biosynthesis to reduce the buildup of these proteins in the ER lumen and to increase the biosynthesis of proteins involved in the machinery for the degradation of proteins associated with the ER (“ER-associated degradation”, ERAD), increase the biosynthesis of chaperone proteins and, finally, help the ER to recover its calcium homeostasis [163,187,188]. This set of signaling pathways, which is a physiological adaptive response of the cell to the accumulation of improperly folded proteins, is called UPR and prevents cell damages and apoptotic mechanisms [189,190,191]. 

Three signaling pathways are involved in the UPR and are initiated by the dissociation of the binding immunoglobulin protein chaperone (BiP) at its luminal part from three effectors, namely, RNA-activated protein kinase-like endoplasmic kinase (PERK), inositol-requiring kinase 1 (IRE1) and activating transcription factor 6 (ATF6) [192,193]. Under physiological conditions, these proteins are transmembrane proteins of the ER and are maintained in an inactive state by the binding of the chaperone protein BiP to their luminal domain. During an ER stress as unfolded/misfolded proteins accumulate in the ER lumen, BiP is released from these complexes in order to activate them. 

The oligomerization of IRE1 induces an autophosphorylation in the kinase domain that allows the X-box binding protein 1 (XBP1) to perform mRNA splicing and form sXBP1, which up-regulates UPR genes [194]. Due to its endonuclease activity, IRE1 also induces degradation of mRNAs localized in the ER membrane in order to decrease their translation by a mechanism called regulated-IRE1 dependent decay (RIDD) [195]. Similar to IRE1, the oligomerization of PERK induces an autophosphorylation and directly phosphorylates the eukaryotic initiation translation factor 2α (eIF2α), which leads to the attenuation of general protein translation. This reduces the ER workload and protects cells from apoptosis by ER stress [196,197]. Paradoxically, the translation of certain mRNAs is increased similarly to the activating transcription factor 4 (ATF4) [198], which plays an important role in the activation of the genes involved in amino acid metabolism, autophagy, antioxidant response and apoptosis. Indeed, ATF4 activates the transcription of target genes encoding the C/EBP homologous protein (CHOP) [199]. Deregulated CHOP activity compromises cell viability and cells lacking CHOP are significantly protected from the lethal consequences of ER stress [200,201]. Furthermore, phosphorylated eIF2α increases the apoptosis antagonizing transcription factor (AATF), that works as a transcription cofactor regulating pro-survival genes under certain conditions of cell stress [202,203,204]. PERK also phosphorylates NRF2, an antioxidant response transcription factor.

Under conditions of ER stress, ATF6 is translocated from the ER to the Golgi apparatus, where it is cleaved at two sites by site-1 (S1P) and site-2 (S2P) proteases [205]. The N-terminal domain of ATF6 migrates to the nucleus where it binds to sequences called endoplasmic reticulum stress response element (ERSE), in order to activate genes encoding ER chaperones, ERAD components and XBP1 [206,207,208].

Due to its location at the ER membrane, *WFS1* has a function in ER homeostasis, more particularly as a negative regulator of the UPR pathway. In physiological condition, *WFS1* plays a crucial role in regulating ATF6α transcriptional activity through HRD1-mediated ubiquitination and proteasome-mediated degradation of ATF6α protein [161]. Indeed, the cleaved form of ATF6 translocates to the nucleus and the non-cleaved form of ATF6 is degraded [209,210]. In ER stress conditions, ATF6α detaches from *WFS1* and regulates stress signaling targets in the nucleus. As ER homeostasis is restored, *WFS1* expression is induced, which results in the degradation of ATF6α [210]. In WS, *WFS1* is not functional due to its mutation. ATF6α is no longer degraded via *WFS1*; therefore, it is hyperactivated regardless of emergency stress conditions, leading to the death of pancreatic β cells. *WFS1*-deficiency also attenuates the AATF–Akt1 pathway and might be involved in the observed β-cell death [204]. Finally, it was seen that, in SH-SY5Ycells transfected with an XBP1-expressing vector, the *WFS1* gene is overexpressed indirectly through an ERSE-like sequence in its promoter and by XBP1 [170]. Indeed, when XBP1 is present in excess, *WFS1* can lower the levels of XBP1S, thus regaining ER homeostasis. Therefore, the role of *WFS1* in the regulation of ER stress revealed some issues, but studies must be carried out to understand its global implication in this cellular protection mechanism. 

The characterization of the three mutant zebrafish lines highlights deficits in some ER stress pathways as a function of the loss of *WFS1* functionality [185,186]. The expression of the different protein factors involved in the three major ER stress pathways (IRE1, PERK and ATF6) were studied by quantitative polymerase chain reaction (qPCR) on 5 dpf zebrafish larvae in basal condition or after induction of ER stress using tunicamycin (2 μg/mL for 24 h at 4 dpf), which induces ER stress indifferently through the three ER stress pathways blocking N-linked glycosylation with transfer inhibition of UDP-N-acetylglucosamine to dolichol phosphate in the ER of eukaryotic cells, thus disrupting protein maturation [211,212,213]. 

The *WFS1a^C825X^* line showed, in basal condition, a decrease in *bip* and *atf4α* mRNA levels and a decrease in hsp90b1 and *chop* mRNA levels in ER stress condition [185], suggesting that only the PERK pathway is impacted after *WFS1a* invalidation. However, the *WFS1b^W493X^* line showed decreased *bip*, *ire1* and *xbp1s*, compared to controls in basal conditions. After tunicamycin treatment, altered increases in *bip*, *ire1*, *perk*, *xbp1s*, *xbp1us*, *eIf2* and *chop* levels were noted [185], clearly showing alteration in the IRE1 and PERK pathways in the mutated *WFS1b* line, while the Atf6 pathway remained unaffected. Interestingly, the *WFS1ab^KO^* line only presented marked alterations in *bip* and *hsp90b1* levels, suggesting that ER stress detection may be altered but the UPR remains functional after complete invalidation of *WFS1*a and *WFS1*b in zebrafish [186] (Figure 1).

Therefore, the *WFS1b^W493X^* line is the most adequate line to mimic the alteration of ER stress response found in WS, as the UPR response is consistently impacted on both the IRE1 and PERK pathways (Figure 1).

Notably, zebrafish offer the possibility to visualize the activation of ER stress in vivo. Indeed, transgenic animals were constructed in order to detect the activation of the different signaling pathways. Concerning the IRE1 pathway, a transgenic zebrafish expressing a part of the cDNA of xbp1 fused to GFP was generated, thus allowing the splicing of xbp1 after the activation of IRE1. This led to the production of a xbp1-GFP protein [214] that allowed the IRE1 pathway to be monitored. Concerning the ATF6 pathway, a transgenic zebrafish expressed five repeated ATF6 consensus binding site upstream of a minimal *c-fos* promoter driving *eGFP* or *d2GFP*. Therefore, when ATF6 is activated, it binds to its binding sites and activates the production of eGFP [215]. Finally, concerning the PERK pathway, a transgenic zebrafish expressing a human ORF*^CHOP^* fused to GFP. Therefore, when PERK is activated, ATF4 expression is induced and CHOP-GFP is produced, allowing the PERK activated pathway to be visualized [216].

### 3.4. WFS1 and Ca^2+^ ER Homeostasis

Mitochondria are complex intracellular organelles, responsible for ATP production, as well as various metabolic cofactors (NADH and FADH_2_), and are involved in different processes such as communication, differentiation, apoptosis and regulation of the cell cycle. Mitochondrial dysfunction has been linked to many neurodegenerative disorders such as Alzheimer’s disease, Parkinson’s disease, amyotrophic lateral sclerosis or Huntington’s disease, which are disabling and often fatal [217,218,219]. The inositol 1,4,5-trisphosphate receptor (IP3R) is responsible for Ca^2+^ release from the ER to the mitochondria, particularly at ER–mitochondria junctions, called MAMs, for mitochondria-associated membranes [220] and composed of a large number of proteins ensuring their structure and functionality [221]. Once Ca^2+^ is released from the ER, it enters mitochondria through the voltage-dependent mitochondrial transmembrane anion channel (VDAC1), whose permeability is controlled by ATP and other regulatory factors [222]. The IP3R/VDAC1 complex is stabilized by the molecular chaperone glucose-regulated protein 75 (GRP75) [223]. Series of chemical reactions are essential to maintain a robust amount of ATP and metabolic intermediates or building blocks for the generation of fatty acids, amino acids and nucleotides, allowing the cells to enter the cell cycle, proliferate and keep normal homeostasis.

The neural Ca^2+^ sensor-1 (NCS1) has been reported to regulate the IP3R [224]. It is a small protein (22 kDa) with four EF-hand motifs (including three of which that bind to Ca^2+^), essential for the release of neurotransmitters [225], synaptic plasticity [226,227], learning and memory [226,228], neurite growth [229] and neuronal survival [230]. In physiological condition, *WFS1* interacts with NCS1 [231,232] and prevents NCS1 degradation by binding to it and forming a complex with IP3R to activate ER-mitochondria Ca^2+^ transfer. When the *WFS1*/NCS1/IP3R complex and VDAC1 are functional, Ca^2+^ can properly transfer from the ER to mitochondria and activate the TCA cycle and mitochondrial respiratory chain. In WS, *WFS1* is no longer functional and the complex *WFS1*/NCS1/IP3R loses its effectiveness, leading to NCS1 degradation and a decrease in ER–mitochondria Ca^2+^ transfer. The MAM fraction is a potential therapeutic target because many neurodegenerative diseases and, more precisely, those of WS, which interest us in this study, have a deficit of MAM, as well as significant ER stress.

A significant amount of information regarding calcium signaling during development in animal models has come from studies on zebrafish [233]. Indeed, the zebrafish embryo is transparent and small and ex utero maturation facilitates the visualization of calcium signals within the whole organism. In addition, its availability of genetically encoded calcium indicators and light-sheet microscopy allowed Ahrens et al. [234] and Panier et al. [235] to image the activity of large numbers of neurons in the brains of zebrafish. The advances in imaging technology [234,236], the processing of the generated data [237,238] and the engineering of encoded calcium indicators [239], allow the activity of a large number of cells on a whole and living organism, such as activation of neuronal circuits in zebrafish, drosophila and mouse, to be measured [240,241].

According to the principle of electrical excitation cells, calcium influx is increased via voltage-gated calcium ion channels, which can be monitored and made visible by calcium imaging using fluorescent calcium probes.

Interestingly, we could imagine, for our study, to analyze the impact of *WFS1* mutation on calcium influx in different cell types affected by WS. By the principle of the GCaMP and Gal4 system widely used today in zebrafish [242,243], it would be enough to create transgenic fish expressing the modified yeast transcription factor Gal4 in specific cell types that degenerate in WS patients. Thus, once the deficit has been measured on these different cell types, testing molecules that would potentially restore these calcium pathways and in fine restore cellular calcium homeostasis.

Similar to the analysis of ER stress, Ca^2+^ imaging is feasible in vivo in zebrafish due to the availability of different transgenic zebrafish expressing GcAMP. Notably, different lines were created in D. Raible’s lab to analyze the Ca^2+^ variation following IP3R stimulation in the ER, cytoplasm and mitochondria of the hair cells of the lateral line [244].

## 4. The Dravet Syndrome (DS)

### 4.1. Physiopathology of DS

**Figure 2:**
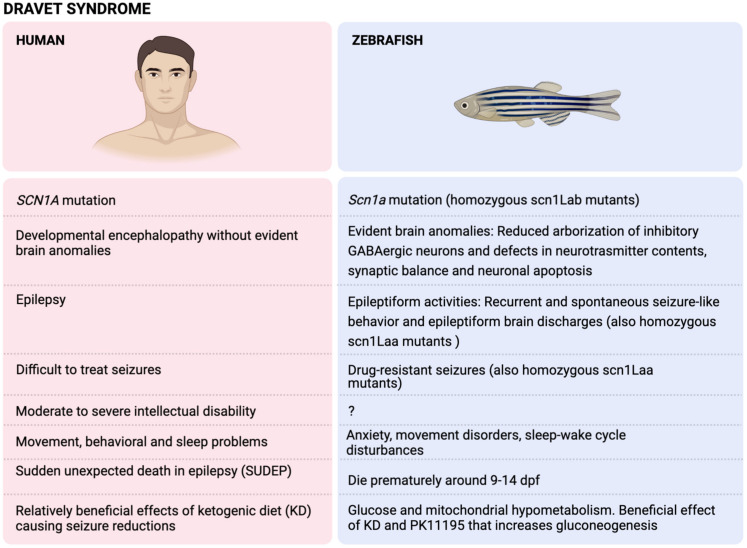
*Scn1a* mutant zebrafish as useful tool to decipher physiopatological deficits induced in Dravet syndrome patients. The SCN1A gene mutation causes Dravet syndrome, which is correlated with several symptoms and is referred to as a “developmental encephalopathy with epilepsy”. In zebrafish larvae (from 3 dpf onwards), these deficits are mostly reproduced; therefore, they mimic human pathology, thus making them a good study model for Dravet syndrome. Adapted from references [117,131,132,135,138,260,264,265,266,267].

Dravet syndrome (DS) is one of the most severe epilepsy syndromes and accounts for up to 6% of the epilepsy cases with onset during infancy. It is named after Dr Charlotte Dravet, who first described this syndrome in 1978. This syndrome is highly characterized by drug-resistant seizures, several physical, intellectual and behavioral comorbidities and a relatively high mortality rate [245] (Figure 2).

#### 4.1.1. Genetics

Almost 90% of DS patients carry a SCN1A mutation, which is also the most prominent epilepsy gene in general. The SCN1A gene codes for the Nav1.1 sodium ion channel, which is expressed throughout the central nervous system (CNS). Even though most genetic mutations are de novo, it is highly recommended for parents to have genetic counseling and genetic examination due to the possibility of parental mosaicism. Regarding genotype–phenotype correlations, truncating mutations seems to be associated with more severe phenotypes than missense mutations. However, the genotype does not appear to be useful in clinic to predict prognosis or choose the proper therapy [245].

#### 4.1.2. Features

During the first year of life, generalized and unilateral seizures, often related to fever episodes, occur in an otherwise healthy child [246,247,248]. These seizures can be clonic or tonic–clonic and are later associated with myoclonus, focal seizures and atypical absences [249]. In the second or third year of life, a decline in neurodevelopmental abilities can be noted and this becomes more evident in adolescence [250]. Other comorbidities can be evident, such as motor problems (e.g., ataxia and gait disturbances) and sleeping problems [251]. Therefore, it is not surprising that the quality of life (QoL) is relatively lower in DS children [245]. Furthermore, the mortality rate is relatively high, with sudden unexpected death in epilepsy (SUDEP) as major cause (>60% of the cases) in teenagers [252] or early adulthood [253].

#### 4.1.3. Brain Anomalies

Brain magnetic resonance imaging (MRI) studies in DS patients usually do not show any anomalies and MRI appears to be normal at epilepsy onset [245,254]. Nevertheless, in a small minority of the patients, structural brain anomalies can be found, e.g., cortical dysplasia, cerebral atrophy, or hippocampal sclerosis [255]. In addition, Lee and colleagues [256] found a reduction in several brain structures, such as gray and white matter, as well as in cerebellar white matter, subcortical volumes and mean cortical thickness. Furthermore, thinning of the corpus callosum, nodular heterotopia and cerebral, cerebellar and hippocampal atrophies were reported in a few DS adolescents [250]. The origin of these brain anomalies is uncertain and does not seem to correlate with epilepsy duration or severity. It is possible that the dysfunctional SCN1A gene confers a unique vulnerability to the brain, which should be investigated in prospective human studies [250,255] and animal models of DS (see Section 4.2.3).

### 4.2. Modelling of DS in Zebrafish (Scn1a Mutants)

Zebrafish *scn1a* mutants (homozygous *scn1lab^−/−^* mutant zebrafish larvae; hereafter referred to as DS zebrafish) were first identified by Schoonheim et al. [257] in an ENU mutagenesis screen. They named these mutants double indemnity (didy; didy^s552^ mutants) zebrafish and described a defect in saccades during optokinetic responses. Three years later, Baraban et al. [138] described an epileptic phenotype in these *scn1lab^−/−^* mutant zebrafish, which are now established as the zebrafish model of DS [126,131,135,258,259]. 

#### 4.2.1. Genetics

The aforementioned DS zebrafish carry two alleles with a point mutation (AG3632G). This mutation leads to the conversion of a thymine (AT3632G, wildtype) into a guanine (AG3632G, mutant), which transforms a methionine into an arginine. Subsequently, this transformation results in a loss of function, similar to the situation in humans with DS [260]. Even though most DS patients are heterozygous for the SCN1A mutation, only the homozygous *scn1lab^−/−^* mutant zebrafish larvae mimic DS features. This apparent discrepancy might be due to the teleost whole genome duplication [261] that resulted in two zebrafish genes homologous to SCN1A, *scn1laa* and *scn1lab*. Therefore, a homozygous mutation in one of these genes equals to the heterozygous state in DS patients. The epileptic phenotype of these stable didy mutants was also observed in a transient zebrafish model of DS by an MO knockdown (KD) of the *scn1lab* gene. Moreover, using this MO KD model, we were the first to show the efficacy of fenfluramine (FA) [262], which is now approved for the treatment of DS patients [263]. FA’s efficacy in DS treatment was also confirmed in stable didy mutants [131,258]. Additionally, another stable mutant was created, i.e., homozygous *scn1laa^−/−^* mutants, that show a similar epileptic phenotype as homozygous *scn1lab^−/−^* mutants [132]. 

Interestingly, *scn1lab* shares 76% identity to the human SCN1A, while *scn1laa* only shares 67% identity [268]. Moreover, homozygous *scn1lab^−/−^* mutants can phenotypically be distinguished from wildtype by their darker appearance, the absence of a swim bladder and a slight body curvature [138], which is not the case for homozygous *scn1laa^−/−^* mutants. Thus, this relatively higher identity percentage and distinct phenotype has boosted the use of *scn1lab^−/−^* mutants as a zebrafish DS model [117,142,145,250,264,269,270,271]. The reason of this darker pigmentation could be the upregulation of melanocortin 5a, although the exact meaning of this upregulation remains unknown [138]. 

#### 4.2.2. Features

Similar to humans, DS zebrafish exhibit recurrent and spontaneous seizure-like behavior (locomotor) and epileptiform brain discharges (LFP) from 3 dpf onwards, until they die prematurely around 9–14 dpf (Figure 2). 

Several genes appeared to be up- and downregulated in DS zebrafish, although many of the identified genes did not have an evident CNS-related expression and/or function [138]. In addition, the downregulation of five glycolytic genes, a significant decrease in baseline glycolytic rate and oxygen consumption rate have been observed in DS zebrafish, suggesting that glucose and mitochondrial hypometabolism might contribute to DS pathogenesis [269,270]. Furthermore, Grone et al. [265] confirmed DS comorbidities in DS zebrafish, such as anxiety, movement disorders and sleep–wake cycle disturbances. 

#### 4.2.3. Brain Anomalies

Further research into the mechanistic features of the loss of function of SCN1A is possible thanks to different DS animal models. DS zebrafish have dynamic changes in progenitor cell and glial population in the CNS and show a reduced arborization of inhibitory GABAergic neurons [117]. Not only GABA, but also other neurotransmitters, such as serotonin (5-HT) and the excitatory neurotransmitter glutamate, could play a role in DS pathogenesis [131,135]. Likewise, there appears to be a defective synaptic balance between excitation and inhibition in DS zebrafish, as well as increased apoptosis of neurons [264]. The exact meaning of these CNS anomalies deserves further research and could boost ASM discovery in these DS zebrafish. For example, the pck1 activator PK11195 increases gluconeogenesis and reduces seizures in DS zebrafish [269] (Figure 2).

### 4.3. DS Zebrafish Mimicking Drug-Resistant Seizures

The spontaneous seizures of DS zebrafish do not respond sufficiently to numerous ASM, although valproate and several GABAergic compounds, e.g., benzodiazepines and stiripentol, are effective [138]. This poor response to ASM is comparable to the drug-resistant seizures in DS patients [272]. Following the guidelines of the North American consensus panel and the European expert group, clinicians should consider valproate as the first ASM in DS. If no sufficient seizure reduction has been obtained, add-on treatment by ASM include topiramate, stiripentol with or without clobazam, cannabidiol, or FA [244]. Even though DS patients are usually treated by a combination of ASMs, we are presently the only zebrafish group confirming the efficacy of combinatorial ASM treatment in DS zebrafish [250], while others only tested one ASM at a time [138,140] (Figure 2). 

### 4.4. DS Zebrafish for Drug Discovery

Due to the drug-resistant nature of seizures in DS zebrafish, effective compounds in this model could not only be effective for treating DS but also seizures related to other severe epilepsy syndromes. For instance, FA was not only active in DS patients but also showed great potential for treating seizures in patients with Lennox–Gastaut syndrome [273] and Sunflower syndrome [274]. 

Before testing any compound in zebrafish, one should determine the maximum tolerated concentration (MTC), since a toxic or lethal concentration could falsely be reported as a seizure-reducing agent. The MTC can be defined as the maximum concentration for which 12 out of 12 zebrafish larvae do not exhibit any signs of toxicity after a 48 h treatment. These toxicity signs can be observed under the microscope, such as a decreased or absent touch response, body deformations, edema, posture loss, anomalies in heart rate or circulation and death [131]. If compounds are randomly tested at arbitrarily chosen concentrations, as done by other groups [138], this could lead to false positives (Figure 2).

#### 4.4.1. Trazodone, TCB-2 and Lisuride

The first drug screening in DS zebrafish by Baraban’s laboratory led to the discovery of clemizole and their radioligand agonist assays showed that clemizole likely is a 5-HT_2A_ receptor (5-HT_2A_R) and 5-HT_2B_R agonist [132]. In striking contrast, they investigated the antiseizure effects of trazodone, which is a 5-HT_2A_R antagonist, thus not an agonist [275,276]. Our research study using the same DS zebrafish—but with a fundamentally different and validated protocol—has shown that 5-HT_2A_R agonists are effective seizure-reducing agents. We used the highly selective 5-HT_2A_R agonists, TCB-2 [131] and NBOH-2C-CN [135], instead of non-selective compounds such as trazodone that also affect other receptors and transporters [266]. Even though TCB-2 could be hallucinogenic—thus, not appropriate as an ASM candidate—novel insights revealed a biased 5-HT_2A_R phosphorylation in response to hallucinogenic versus non-hallucinogenic agonists [277]. This finding paves the way to repurpose non-hallucinogenic 5-HT_2A_R agonists for DS treatment, such as lisuride [126], which has already been safely used to treat pediatric and adult patients with Parkinson’s disease, migraine and cortical reflex myoclonus [267,278,279,280]. 

Since GABAergic interneurons show severe impairments in DS pathophysiology [281,282], the modulation of GABAergic neurotransmission could play a role in DS treatment. Of importance, 5-HT_2A_R agonism seems to facilitate GABAergic neurotransmission, which could explain the efficacy of 5-HT_2A_R agonism for DS treatment [283] and other forms of epilepsy [284,285,286]. While these studies highlight the potential of 5-HT_2A_R agonism in epilepsy treatment, others have shown that 5-HT_2A_R stimulation could even reduce SUDEP [287,288,289]. Buchanan and colleagues demonstrated that the aforementioned 5-HT_2A_R agonist, TCB-2, not only reduces seizures but also lowers seizure-induced mortality in Lmx1^bf/f/p^ mice—that lack over 99% of CNS 5-HT—in acute pilocarpine and maximal electroshock seizure (MES) models. Overall, a plethora of research works underline the beneficial effects of 5-HT_2A_R agonism in treating epilepsy and potentially reducing SUDEP. Therefore, the questionable findings of trazodone, a 5-HT_2A_R antagonist, should be considered carefully.

#### 4.4.2. Lorcaserin

We were the first to discover that lorcaserin, a 5-HT_2C_R agonist, significantly reduced abnormal behavioral and electrographic seizure activities in DS zebrafish [131]. Subsequently, these results were replicated by Griffin et al. [132], underlining that serotonergic modulation as a pharmacological modality for treating DS was not novel at that time.

Regarding the potential efficacy of this compound in five DS patients, the rather small non-placebo controlled clinical study is difficult to interpret since the exact seizure frequencies are not provided [132]. After three months of treatment, nearly all (four out of five) patients returned to their baseline seizure frequency and only two patients remained on the drug [290]. Therefore, these findings are too preliminary and larger trials are warranted. Nonetheless, ample evidence is available indicating that 5-HT_2C_R agonists are interesting compounds for treating effectively neurological diseases such as epilepsy [291,292,293,294,295], if they do not stimulate 5-HT_2B_R [296].

#### 4.4.3. Clemizole and Analogs Stimulating 5-HT_2B_Rs

The 5-HT_2B_Rs are mainly located in the heart and 5-HT_2B_R agonists could lead to drug-induced cardiotoxicity [297,298,299]. That is why repurposing lisuride for DS treatment holds great promise, since it significantly reduces seizures in the DS zebrafish model [126], acts as a 5-HT_2B_R antagonist and thereby is devoid of any cardiotoxic effects [280]. 

Baraban and colleagues showed the efficacy of clemizole, a 5-HT_2B_R agonist and other 5-HT_2B_R agonists in DS zebrafish [138]. Whereas 5-HT_2B_R agonists were ineffective in the same zebrafish model [131,135]. Two independent research groups [259,271] showed that clemizole was toxic at the concentrations used by Baraban’s lab [140]. After a toxicity assessment, they have tried clemizole at lower concentrations and discovered that this compound does not reduce seizures [259,271]. 

#### 4.4.4. Fenfluramine

Based on our DS zebrafish research studies, FA is likely to act via the 5-HT_1D_R, 5-HT_2C_R and σ_1_ receptors [135]. FA’s seizure reduction was not counteracted by 5-HT_2B_R antagonism, indicating that 5-HT_2B_R agonism is not responsible for the efficacy of this FDA- and European Medicines Agency (EMA)-approved drug for treating DS. Inevitably, these findings suggest that 5-HT_2B_R agonism is not necessary to treat seizures in DS patients. The weak agonistic activity of FA at 5-HT_2B_Rs could be responsible for drug-induced valvulopathy, although severe FA-induced cardiotoxicity was only observed when this drug was used as a weight-loss agent at much higher doses and/or in combination with other amphetamine-like drugs [263,297,300,301]. For this reason, clinical trials with low-dose FA monitor cardiac side effects closely and, until now, FA’s safety has been guaranteed [302]. 

Equally important, our DS zebrafish research studies demonstrated the efficacy of a highly selective 5-HT_1D_R agonist, GR 46611, which was confirmed in a DS mice model and even expanded the lifespan in these DS mice [303]. Therefore, one might believe that 5-HT_1D_R agonistic activities of FA can significantly reduce mortality in DS.

Others have shown that 5-HT_2A_R [304] and 5-HT_4/7_R [305] are also involved in FA’s mechanism of action and potentially reduce SUDEP [305,306]. Finally, FA can even restore the neuronal cytoarchitecture in DS zebrafish on a cellular level; thus, FA could be an anti-epileptogenic compound [117].

In clear contrast to the antiseizure effects of clemizole that were, up to now, only validated by one zebrafish research group [138], FA has been proven to be an effective ASM in several animal models of epilepsy (rodent and zebrafish) by multiple researchers worldwide [117,131,140,271,305]. 

Overall, FA is likely a multidimensional ASM via 5-HTR agonism, positive allosteric modulation of σ_1_ receptors and maybe other unidentified pathways [307,308,309]. Taken together, these preclinical data in DS zebrafish provide new avenues for ASM discovery and warrant further exploratory studies in mice and PWEs. 

## 5. Pros and Cons of Zebrafish Models in a Context of Molecules High-Throughput Screening

**Figure 3:**
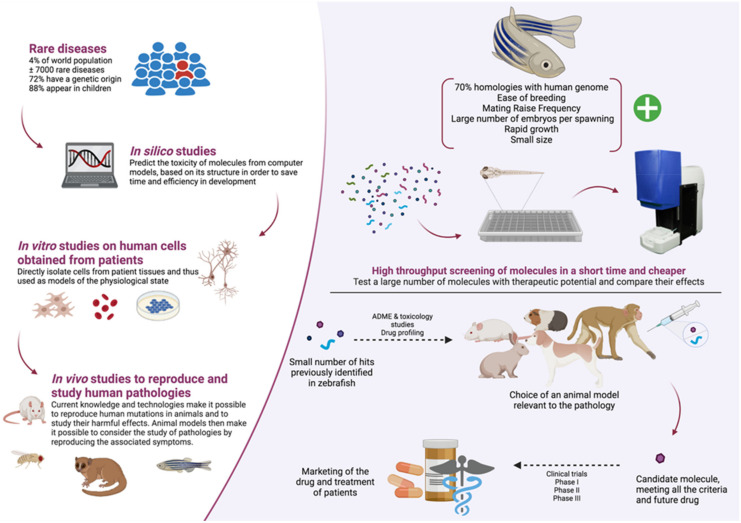
Benefits from the high throughput in vivo screening in zebrafish for the discovery of novel or repositioning drugs in rare genetic diseases. The zebrafish model speeds up studies of rare human diseases to find active molecules to treat associated deficits. The development of a potential future drug involves several stages, often long and expensive. Zebrafish, a useful model that mimics most human pathologies, have shown many advantages for drug development compared to other animal models used.

The use of zebrafish as a powerful model to screen drugs in vivo has gained momentum in the past two decades. This small vertebrate organism has emerged as an intermediate model of choice between the low-throughput and costly rodent models and the cellular models, which allow for high-throughput screening to be performed but lack physiological context (Figure 3).

The keen interest in this little fish can be explained by its inexpensive husbandry associated with its high fecundity and the easiness of its maintenance, favoring large-scale screening. Due to their small size, they can be housed, as adults, in large numbers, but, more importantly, embryos and larvae can be housed in 96-well or 384-well plates, prerequisite for high-throughput analysis protocols.

Drug-induced morphological and developmental defects can be easily experimentally tracked thanks to the transparency of the embryos and the external development of the larvae. In addition, many neurobehavioral phenotypes, such as sleep, addition, learning, vision and hearing, among others, can be quantified using different tracking devices, as described previously, facilitating the study of a given phenotype. In addition, the anatomical and physiological features of zebrafish are relatively similar to the ones in humans. Therefore, zebrafish can be used as a relevant model to study the impact of a given drug on a specific structure or organ.

With the advent of genetic analyses, including next-generation sequencing, a large number of genetic disease causes have been identified, including genes and variants associated with rare diseases [310,311]. Concomitantly, the genetic sequences of the zebrafish genome were published and made accessible [4]. The recent emergence of tools to manipulate the genome combined with the knowledge of both the disease-leading genes in human and the sequences of their zebrafish orthologues is a powerful methodology to create disease-specific models. Zebrafish can be genetically engineered to mimic human mutations, in transient gain or loss of function models (mRNA injection, morpholinos) as well as stable mutant lines (TALENs, CRISPR/Cas9). These methods have been optimized in zebrafish and they have proven to be fast and cost-effective, humanized fish being thus extensively used for targeted drug screening.

Even though zebrafish have emerged as a powerful animal model to study physio-pathology of human diseases, some limitations need to be taken into account. Zebrafish are vertebrates and share a lot of morphological similarities with human, as described previously. However, it is crucial to acknowledge that some anatomical divergences may modify the development of the disease in this model, as well as the impact of the tested drugs. As an example, zebrafish do not have a dedicated auditory organ comparable to the inner ear in mammals. The difference was thus circumvented, efficiently, by the study of the hair cells from the lateral line [312]. Therefore, it is important to consider these differences when choosing the zebrafish as an animal model for a given rare disease.

In addition, even though 70% of human genes have an orthologous gene in zebrafish [4], modifying the expression of these genes to mimic human pathology may lead to a phenotype that would differ from the human one. The analysis would complexify for the 20% genes that have two orthologs [4]. The duplication of these genes can impede the efficiency of forward or reverse genetic approaches.

Lastly, pharmacokinetic is not as well characterized in zebrafish as it is in mammals. While tremendous efforts have been made recently and the accumulation of pharmacokinetic studies have unraveled new insights (e.g., [313,314]), to pave the way to a more comprehensive understanding of zebrafish pharmacology, more studies are needed. A better understanding of the pharmacokinetics would help optimize the screening protocols as well as allowing researchers to conduct a rapid transfer to mammal models.

## Figures and Tables

**Table 1:** Different approaches to assess brain activity in zebrafish larvae. Abbreviations: LFP, local field potential; iZAP, integrated Zebrafish Analysis Platform.

	LFP	iZAP	Multi-Electrode Array	Bioluminescence
Number of zebrafish larvae	1	Multiple	1	Multiple
Possible recording time	Hours	Hours	Days	Days
Areas of neuronal activity	1	4 (brain, muscle, eye and ear)	61	Multiple

## Data Availability

Not applicable.

## Abbreviations

5-HT	serotonin
5-HTR	serotonin receptor
AATF	apoptosis antagonizing transcription factor
ASM	anti-seizure medication
ASR	acoustic startle response
ATF4	activating transcription factor 4
ATF6	activating transcription factor 6
BIP	glucose-regulated protein (GRP-78)
CHOP	C/EBP homologous protein
CRISPR	clustered regularly interspaced short palindromic repeats
DEE	developmental and epileptic encephalopathies
DIDMOAD	diabetes insipidus, diabetes mellitus, optic atrophy, deafness
dpf	day post fertilization
DS	Dravet syndrome
EKP	ethyl ketopentenoate
EMA	European Medicines Agency
ENU	N-ethyl-N-nitrosourea
ER	endoplasmic reticulum
ERAD	endoplasmic reticulum-associated degradation
FA	fenfluramine
GABA	γ-aminobutyric acid
GCL	ganglion cell layer
GFP	green fluorescent protein
GRP75	glucose-related protein 75
ILAE	International League Against Epilepsy
INL	inner nuclear layer
IP3R	inositol 1,4,5-trisphosphate receptor
IRE1	inositol-requiring enzyme 1
iZAP	integrated Zebrafish Analysis Platform
KD	knock-down
KO	knock-out
LFP	local field potential
MAM	mitochondria-associated ER membrane
MES	maximal electroshock seizures
MO	morpholino oligonucleotide
MRI	magnetic resonance imaging
MTC	maximum tolerated concentration
NCS1	neural calcium sensor-1
OKR	optokinetic response
OMR	optomotor response
ONL	outer nuclear layer
ORF	open reading frame
PERK	protein kinase R (PKR)-like endoplasmic reticulum kinase
PTZ	pentylenetetrazole
PWE	patient with epilepsy
qPCR	quantitative polymerase chain reaction
RIDD	regulated-IRE1 dependent decay
RNAi	RNA interference
S1/2P	site 1/2 protease
SCN1A	sodium voltage-gated channel alpha subunit 1
SUDEP	sudden unexpected death in epilepsy
TALEN	transcription activator-like effector nuclease
UPR	unfolded protein response
VDAC1	voltage-dependent mitochondrial transmembrane anion channel
VMR	visual motor response
*WFS1*/2	Wolfram syndrome type 1/2
WS	Wolfram syndrome
XBP1	X-box binding protein 1
